# Water, Its Agency in Therapeutics

**Published:** 1887-05

**Authors:** B. M. Lawrence


### WATER, ITS AGENCY IN THERAPEUTICS.

BY B. M. LAWRENCE, M. D.

Water is the great agent employed, both by nature and art, in all the important processes of growth and purification, and since all forms of disease are caused by or complicated with impurity of some kind, as we have indicated in previous articles, the importance of this element as a curative agent is readily understood. We are therefore not surprised that the renowned Fathers of the healing art, including the most eminent physicians from the days of Hypocrates to Priessnitz, have been outspoken in their praise of Water as a valuable auxiliary in the treatment of disease.

*Galen* placed water in the highest ranks in his *Materia Medica.* *Celsus* advised its use in a score or more of the most obstinate diseases; *Hoffman* pronounced water a "remedy suited to all persons and all times, and to all diseases both acute and chronic, and also that its use, answers all indications, both of prevention and cure".

*Boerhave* left on record that "No remedy can more effectively secure health and prevent disease than pure water." *Haller* as evidence of his appreciation of its value drank nothing but water, and the same is recorded as the habit of *Demosthenese*, *Lock*, and *Milton*, *Lancassani* in 1753, *Caldani* in 1767, *Leanter* in 1780, and *Percy* in 1785, published conclusive evidence of the superiority of water alone to all the medicated fluids and compounds known, for the cure of surgical diseases, and recent experience in the Hospitals during the late war between France and Germany fully sustained their conclusions *Dr. Forbes*, while editing the ablest medical journal in the world, and royal physician to Her Majesty Queen Victoria, said that "in a large proportion of cases of gout and rheumatism the Water Cure seems to be extremely efficacious," and for "a large number of complex diseases usually known as chronic dyspepsia, when all other modes of treatment have failed or been only partially successful, the Practice of Priessnitz is well deserving of a trial". If the object was to cure the patient at once, as it would be if he was employed by the year as we have suggested, would not the doctor use his best aud most succesaful mode of treatment, before "all other modes have failed."

These eminent practitioners of the regular medical schools are only a fragment of the multitude who have left on record the strongest testimony in favor of water as one of our most beneficial agents in the cure of disease. In order to understand the *modus operandi* of the water treatment or the relation of pure water to morbid conditions, it becomes necessary to comprehend its vital relation to the system in a normal or healthful condition, as stated in the following distinct propositions which can be easily remembered.

1. Water constitutes more than one-half of the entire bulk of the body.

2. Water composes more than three-fourths of the whole mass of the blood; more than seven-eighths of the brain, and more than nine-tenths of the saliva and other colorless secretions.

3. Water is the only vehicle by which nutrient matters are conveyed to the blood, and through it to all parts of the body, bearing the elements of growth and repair.

4. Water is the only medium through which the waste or worn out particles are conveyed from all parts of the body to the excretory organs to be expelled.

5. Water is the only solvent with which to dilute and prepare for exit from the system the refuse matter in the alimentary canal, etc.

6. Water is the only material capable of circulating in all the finest blood vessels and tissues without causing injury or irritation.

7. Water never produces morbid effects or ill results except from its being used at an improper temperature or from its use in excessive quantities, effects by no means unavoidable.

The *rationale* of the water treatment which has already—since the days of Priessnitz—greatly modified and improved the medical practice of every school, will be readily understood, when we remember that diseases are produced by bad air, improper light, impure food and drink, excessive or defective alimentation, indolence or over-exertion, unregulated passions, in three words unphysiological voluntary habits. The conditions of the body in disease, the proximate causes against which all remedial efforts are to be directed are, in general terms, impure blood, unhealthy recreations, obstructions in the minute vascular structures, or capillary vessels, excessive action in some parts or organs, deficient action in others, unequal temperatures, in other words, a loss of balance in the circulation, and action of the various parts of the vital mechanism, producing great discord in some portions of it, and more or less disorder in all. The general indications are therefore, to remove obstructions, wash away impurities, supply healthy nutriment, regulate temperature, relax intensive and intensify torpid action, etc., and what like water and what but water, with its concomitants oxygen, light, food, heat or temperature, etc., can answer to these indications. To say that medicinal drugs can answer these indications is sheer nonsense; they may respond to almost any other indications, but to these never. The further uses of water for bathing, etc., will be considered in future articles.

